# The Relative Reinforcing Value of Cookies Is Higher Among Head Start Preschoolers With Obesity

**DOI:** 10.3389/fpsyg.2021.653762

**Published:** 2021-04-30

**Authors:** Sally G. Eagleton, Jennifer L. Temple, Kathleen L. Keller, Michele E. Marini, Jennifer S. Savage

**Affiliations:** ^1^Department of Nutritional Sciences, College of Health and Human Development, The Pennsylvania State University, University Park, PA, United States; ^2^Center for Childhood Obesity Research, College of Health and Human Development, The Pennsylvania State University, University Park, PA, United States; ^3^Departments of Exercise and Nutrition Sciences and Community Health and Health Behavior, School of Public Health and Health Professions, University at Buffalo, Buffalo, NY, United States; ^4^Department of Food Science, The Pennsylvania State University, University Park, PA, United States

**Keywords:** reinforcing value of food, obesity, energy density, children, low-income, food insecurity

## Abstract

The relative reinforcing value (RRV) of food measures how hard someone will work for a high-energy-dense (HED) food when an alternative reward is concurrently available. Higher RRV for HED food has been linked to obesity, yet this association has not been examined in low-income preschool-age children. Further, the development of individual differences in the RRV of food in early childhood is poorly understood. This cross-sectional study tested the hypothesis that the RRV of HED (cookies) to low-energy-dense (LED; fruit) food would be greater in children with obesity compared to children without obesity in a sample of 130 low-income 3- to 5-year-olds enrolled in Head Start classrooms in Central Pennsylvania. In addition, we examined individual differences in the RRV of food by child characteristics (i.e., age, sex, and reward sensitivity) and food security status. The RRV of food was measured on concurrent progressive-ratio schedules of reinforcement. RRV outcomes included the last schedule reached (breakpoint) for cookies (cookie Pmax) and fruit (fruit Pmax), the breakpoint for cookies in proportion to the total breakpoint for cookies and fruit combined (RRV cookie), and response rates (responses per minute). Parents completed the 18-item food security module to assess household food security status and the Behavioral Activation System scale to assess reward sensitivity. Pearson’s correlations and mixed models assessed associations between continuous and discrete child characteristics with RRV outcomes, respectively. Two-way mixed effects interaction models examined age and sex as moderators of the association between RRV and Body Mass Index z-scores (BMIZ). Statistical significance was defined as *p* < 0.05. Children with obesity (17%) had a greater cookie Pmax [*F* (1, 121) = 4.95, *p* = 0.03], higher RRV cookie [*F* (1, 121) = 4.28, *p* = 0.04], and responded at a faster rate for cookies [*F* (1, 121) = 17.27, *p* < 0.001] compared to children without obesity. Children with higher cookie response rates had higher BMIZ (*r* = 0.26, *p* < 0.01); and RRV cookie was positively associated with BMIZ for older children (5-year-olds: *t* = 2.40, *p* = 0.02) and boys (*t* = 2.55, *p* = 0.01), but not younger children or girls. The RRV of food did not differ by household food security status. Low-income children with obesity showed greater motivation to work for cookies than fruit compared to their peers without obesity. The RRV of HED food may be an important contributor to increased weight status in boys and future research is needed to better understand developmental trajectories of the RRV of food across childhood.

## Introduction

In the United States, changes in the environment, which have facilitated greater expression of obesity-related genes at a population level, are largely responsible for the obesity epidemic (Novak and Brownell, 2011). The current food environment promotes positive energy balance (Jeffery and Utter, 2003) due to easily accessible and abundant highly palatable, energy dense foods that, compared to healthier options, are cheaper and require minimal effort to obtain (Drewnowski, 2004; Novak and Brownell, 2011). Human brain circuitry is hard wired to respond to foods high in calories, sugar and fat (King, 2013). One factor that may contribute to excess energy intake in our modern food environment is the relative reinforcing value (RRV) of food, or motivation to eat, defined as how hard an individual will work to access a food when an alternative food or non-food reward is concurrently available (Epstein et al., 2007). Low-income children are disproportionately affected by obesity. On average, 23% of United States preschoolers have overweight or obesity (Ogden et al., 2014) while the prevalence of overweight and obesity among low-income preschoolers has been shown to range from 32 to 35% (Williams et al., 2004; Edmunds et al., 2006; Kimbro et al., 2007). The diets of low-income children are well below national dietary recommendations (Leung et al., 2013) and evidence suggests that among low-income children, those experiencing food insecurity (FI) are exposed to more obesogenic home food environments than their food secure counterparts (Nackers and Appelhans, 2013). The RRV of food is associated with higher weight across childhood (Temple et al., 2008; Rollins et al., 2014b; Kong et al., 2015; McCullough et al., 2017; Vervoort et al., 2017; Wong et al., 2019), yet this relationship has not been examined in low-income preschool-age children. Further, the association between FI and weight status in young children is mixed (Dinour et al., 2007; Eisenmann et al., 2011).

Rollins et al. (2014b) established a modified RRV task suitable for 3- to 5-year-olds in the childcare setting. Results showed that children with higher Body Mass Index z-scores (BMIZ) responded for food at a faster rate. Other studies with this age group reveal that the RRV of high-energy-dense (HED) to low-energy-dense (LED) food and the RRV of HED food to a non-food alternative (e.g., coloring or doing puzzles) are associated with overweight and higher BMIZ, respectively (McCullough et al., 2017; Wong et al., 2019). The generalizability of these findings is limited. First, study samples were relatively small and homogenous—highly educated, middle-to-upper-income. Second, two studies (McCullough et al., 2017; Wong et al., 2019) used sequential designs where children work for each reward one at a time, which may not generalize to the real world where eating typically involves choices between multiple foods (Epstein et al., 2007). Finally, although Rollins et al. (2014b) used a concurrent design, children worked for similar foods [two different shaped HED graham crackers (4.5 calories/gram)], prohibiting the examination of RRV by energy density.

It is also unclear how individual differences in the RRV of food develop across childhood. Child characteristics that have been linked to the RRV of food include age, sex, and reward sensitivity, a temperamental trait implicated in appetitive motivation and obesity risk (Blair, 2003). Two prior studies showed a positive (Rollins et al., 2014b) and null (Vervoort et al., 2017) association between the RRV of food and reward sensitivity. In addition, research examining sex and age differences is limited to a few studies. Research with preschoolers (Rollins et al., 2014b) and adolescents (Vervoort et al., 2017) shows that boys work harder for food than girls, yet a study conducted with school-age children found an association between the RRV of food and BMIZ among girls only (Gearhardt et al., 2017). With regard to age, responding for both food and monetary rewards increases from age three to five (Rollins et al., 2014b) and four to 14 years (Chelonis et al., 2011), respectively. However, it is unknown whether the association between the RRV of food and child weight becomes more pronounced across childhood and additional research on sex differences is needed given the inconsistent findings. Similarly, whether factors in the home environment influence the development of the RRV of food in children is poorly understood. Food deprivation increases the RRV of food (Epstein et al., 2003; Raynor and Epstein, 2003); and although caloric deprivation is no longer common in the United States, limited access to food resulting from FI may lead to increases in food reinforcement (Crandall and Temple, 2018). Two recent studies with adults support this notion (Crandall and Temple, 2018; Crandall et al., 2020). One study showed increases in the RRV of food in response to experimentally manipulated scarcity among food insecure, but not food secure individuals (Crandall and Temple, 2018). Another study conducted with pregnant women found an association between very low food security and higher RRV of food (Crandall et al., 2020). Whether the RRRV of food differs by food security status has not been examined in children.

The current cross-sectional study examined the RRV of HED (cookies; >4 kcal/g) to LED (fruit; <1 kcal/g) food in a sample of low-income 3- to 5-year-olds using a concurrent design in the children’s naturalistic setting (i.e., school). The first objective was to test the hypothesis that children with obesity would have a higher RRV of cookies vs. fruit than children without obesity. The second objective was to examine whether the RRV of food is associated with child characteristics (i.e., age sex, and reward sensitivity) and food security status, and to explore children’s age and sex as moderators of the association between the RRV of food and BMIZ. Based on previous research (Rollins et al., 2014b; Vervoort et al., 2017; Crandall and Temple, 2018; Crandall et al., 2020), we hypothesized that: (1) compared to girls, boys would respond more and at a faster rate for both cookies and fruit, (2) older children would respond more for cookies and fruit, and (3) the RRV of cookies to fruit would be positively associated with BMIZ, reward sensitivity, and FI.

## Materials and Methods

### Participants

Child-caregiver dyads enrolled in Head Start in Central Pennsylvania were recruited from 15 full-day classrooms within eight Head Start centers. Teachers sent recruitment letters and study packets home with all children in participating classrooms at the beginning of fall 2018 (seven classrooms) and spring 2019 (eight classrooms). Study packets included a caregiver survey and a child consent form. The caregiver survey utilized implied consent, and instructions were provided for returning the survey in the mail. Out of 235 study packets distributed across both semesters, 199 caregiver surveys were returned (85%). The child consent form included directions for signing one of two signature lines: one that provided consent for their child’s participation or one that denied consent. Caregiver participation in the survey was not required for consented children to participate in the RRV task or to obtain height and weight measurements (*n* = 213; 91%). Caregivers received a $40 gift card for returning the survey and each participating classroom was compensated with a $100 gift card to purchase supplies for their classroom. The majority of caregiver respondents were parents/legal guardians (94%; 5% grandparents; 1% foster parent) and are referred to as parents hereinafter.

**FIGURE 1:**
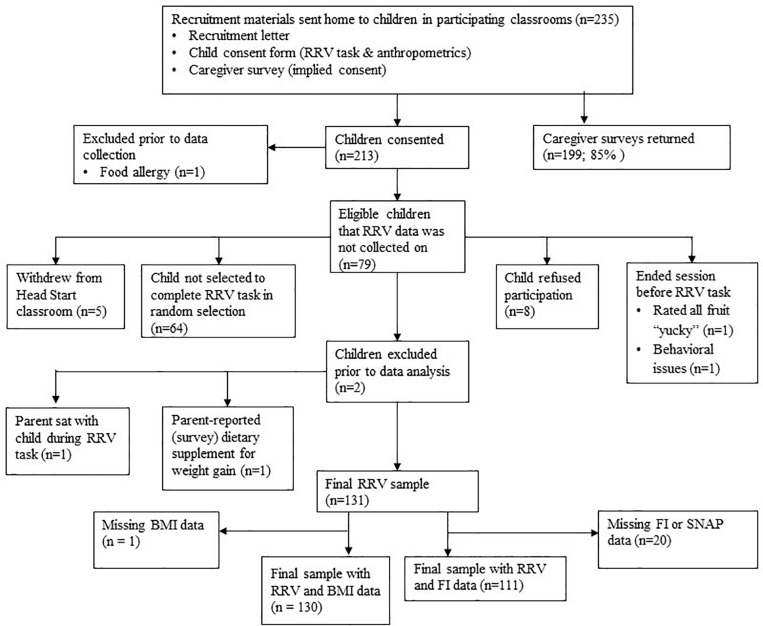
Consort diagram.

The current study was designed to examine the relationship between FI and the RRV of food, which has not been previously studied in children. Prospective power calculations to determine sample size were made based on a study with preschoolers reporting mean differences in the RRV of food by weight status (McCullough et al., 2017). Our power calculations indicated that we would need 48 participants per each of two food secure groups (total of 96), to detect an effect size (Cohen’s d) of 0.58 (McCullough et al., 2017), assuming nominal power of 0.80, and *p* < 0.05. Based on previous data (Na et al., 2020), we anticipated a FI rate between 30 and 40% and five to seven children from FI households per each classroom of approximately 18 children. We also anticipated the possibility of low child enrollment in our study. Thus, to ensure that we reached our enrollment goal of 48 FI children, we aimed to recruit a minimum of 12 classrooms (i.e., ∼140 food secure children and 75 FI children). A consort diagram showing study recruitment, with enrollment statistics, is provided in Figure 1. Resources were limited (e.g., staff time, travel costs) due to higher-than-expected child enrollment during fall 2018. Additionally, as predicted, we had an imbalance in the number of FI participants at the end of fall 2018 (i.e., 25 FI vs. 47 food secure). Thus, to reduce costs and to ensure reasonable representation of the FI group, a weighted selection process was developed and used in classrooms that participated in spring data collection. One research staff member, who did not participate in data collection, examined parent surveys and determined food security status. For each classroom, survey results on food security status were used to categorize consented children as FI or food secure. The same research staff member randomly selected a child from a food secure household for each participating FI child per classroom to ensure that research staff collecting RRV data remained blinded to children’s food security status. Data collection was discontinued in Spring 2019 once all FI children with a completed survey had participated in the RRV task, which resulted in 113 children with food security and RRV data (41% FI; 59% food secure). As a result, 64 consented children were not selected to participate in the RRV task. Head Start teachers provided children’s food allergy information, and one child was excluded prior to data collection due to an allergy to a study food. Children were also dropped from the study because they withdrew from Head Start (*n* = 5), refused to participate in the RRV task (*n* = 8), or had the session terminated prior to the task due to behavioral difficulties (*n* = 1).

### Procedure

Prior to data collection, study staff members visited each participating classroom to familiarize children with the study foods. Study foods were introduced one at a time and children had the opportunity to taste each food. Up to two make-up familiarization days were provided for classrooms in which children were absent at the initial visit. Three participants were absent on all familiarization days. Excluding these three children did not change results, thus they were retained in the current analyses. The RRV task was administered in a study session initiated 60–120 min after children had a typical Head Start provided lunch. In the study session, children completed the hunger assessment, liking assessment, and the RRV of food task. Following completion of the RRV task, children had the opportunity to eat the food portions earned during the task. The hunger assessment was then re-administered, and foods were weighed in order to calculate energy intake. This study was approved by the Office for Research Protections at The Pennsylvania State University, United States.

### Measures

#### RRV of Food Task

**FIGURE 2:**
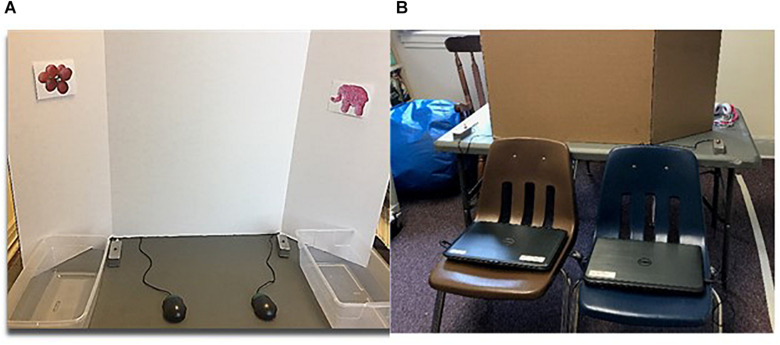
Data collection station for the relative reinforcing value (RRV) of food task. **(A)** Child’s view during the RRV task. **(B)** Computer set-up that is hidden from children’s view.

The RRV of cookies and fruit were assessed using the RRV of food task (Epstein et al., 2007), adapted for young children and suitable for use in the childcare setting (Rollins et al., 2014b). The RRV task was administered to children in individual data collection stations at Head Start centers. Stations were set up following the protocol developed by Rollins et al. (2014b) (Figure 2). With the exception of one classroom that was its own free-standing building, children were removed from their classroom and completed the RRV task in a separate room. To reduce distractions, children were asked, but not required, to wear noise canceling headphones during the task. Children had the option to work to gain access to small portions of cookies on one mouse or small portions of fruit on a second mouse. Children were instructed that pressing the mouse to their right would earn them cookies and pressing the mouse to their left would earn them fruit. A picture of the cookie and fruit was placed above its corresponding mouse and a light was positioned next to each picture that indicated when a food reward was earned. The foods were pre-portioned in clear condiment cups with lids. The three fruits were matched according to the weight of one portion as best as possible; fruit portions: grapes (halved; ∼18.2 g/portion), canned mandarin oranges (∼16.5 g/portion), and canned pineapple (∼17.9 g/portion); as were the weights for the cookies: Oreo minis^TM^ (∼6.4 g/portion), Fudge stripes minis^TM^ (∼7.1 g/portion), and Circus animal^®^, cookies (∼7.1 g/portion). Within food categories, gram weight was balanced to keep calories consistent regardless of the fruit or cookie chosen. Matching across food categories would have resulted in larger portions of fruit compared to cookies. To avoid a potential portion size effect (i.e., clicking more for fruit or consuming more fruit as a function of a size-related visual cue) (Fisher and Kral, 2008), the gram weight was not matched across food categories. The canned fruits were in 100% juice and the juice was drained prior to being portioned out. Upon earning a reward, the pre-portioned food was placed in a clear bin next to its respective mouse. Both mice, which were connected to hidden computers, were on independent, concurrent progressive-ratio (PR) schedules of reinforcement. The PR schedules began at four and doubled each time a reward was earned; this means that a food portion was earned after 4 clicks on the first trial, then 8 clicks on the second trial, and so on, with a maximum of 8 trials (i.e., 4, 8, 16, 32, 64, 128, 256, and 512 clicks). This PR schedule has been used in prior studies with older children and preschoolers (Temple et al., 2008; Rollins et al., 2014b).

Before the RRV task, instructions were given (and repeated back) and children completed a practice round. Children were told that they could only click one mouse at a time, that they could stop earning food at any time, and that they could not eat their food rewards throughout the task but would have the opportunity to eat what they earned after the task. A scripted phrase (i.e., “here’s what you earned. You can earn more [food #1] by pressing the same button. You can earn [food #2] by pressing the other button”) was stated after the first food portion was earned to ensure children’s understanding. Scripted reminders of the rules (e.g., “remember, press this button to earn fruit and this button to earn cookies”) were provided at the beginning of the task if a child looked confused. Children completed the task independently while a research staff member was nearby to provide food portions earned, to answer questions, or when necessary, to remind the child of the rules. Children had up to 20 min to complete the task but could stop prior to 20 min upon indicating they were done. If a child looked as if they had completed the task, they were asked if they were finished and confirmed by asking, “are you done playing”? Next, children were given a 10-min snack session to eat the cookies and fruit that they earned during the task. Cookies and fruit were placed in separate bowls and were weighed pre- and post-snack time to determine the amount consumed (gram weight). Product label information was used to determine the energy density (ED) (kcals/grams) of each food and energy intake was calculated (kcals = ED × grams).

The RRV of food, operationalized as the breakpoint for one food in proportion to the breakpoint for both foods, was calculated for cookies [RRV cookie = cookie Pmax/(cookie Pmax + fruit Pmax)] (Kong et al., 2015). The breakpoint for each food, operationalized as the highest trial in which responses were made, was determined for cookies (cookie Pmax) and fruit (fruit Pmax). The breakpoint reflects the reinforcing value of a reward, and a reward with a higher breakpoint is considered more reinforcing than a reward that a participant stops responding for earlier (Epstein et al., 2007). RRV of cookie greater than 0.5 indicates that more trials were completed for cookies (i.e., had a higher breakpoint for cookies) and that the child was more motivated to gain access to cookies compared to fruit. A mean response rate for each food was calculated by averaging the number of responses (button presses) per minute across all trials (Rollins et al., 2014b).

#### Hunger Assessment

Children’s hunger level before and after the RRV task was measured using a modified version of a protocol that has been used in previous studies with preschoolers (Fisher and Birch, 2002). Children were read a story about a little boy/girl who can see inside his/her tummy. Three pictures were presented ranging from hungry to full, depicted by the child with (1) an empty stomach, (2) a half empty/full stomach, and (3) a full stomach. Children were asked to repeat back which picture showed the child with the empty, half empty/full, and full stomach to ensure children’s comprehension. Next, children were asked to think about how their stomach feels and to indicate their own level of hunger/fullness on a three-point scale using the three pictures of the little boy/girl.

#### Liking Assessment

Children’s liking of the study foods was measured prior to the RRV task using a liking assessment established by Birch (Birch, 1979). Children were first familiarized with three faces visually representing “yummy,” “just okay,” and “yucky.” Each food, pre-portioned for the RRV task in a clear condiment cup, was presented to children one at a time in a pre-selected order. The three fruits (i.e., red grapes, oranges, and pineapple) were presented first followed by the three cookies [i.e., Oreo minis^TM^ (Nabisco Co., East Hanover, NJ, United States), Fudge stripes minis^TM^ (Keebler Co., Battle Creek, MI, United States), Circus animal^®^, cookies (Mother’s Co., United States)]. Children did not taste the foods due to time constraints at schools, but each food was identified before (e.g., “these are grapes”) and during (e.g., “Do you think grapes are yummy, yucky, or just okay?”) the rating for each food. Utilizing the three pictures, children were asked to categorize whether they thought each fruit was “yummy,” “yucky,” or “just okay.” If a child categorized more than one fruit as “yummy,” they were asked to choose the “yummiest.” If none of the fruits were categorized as “yummy,” children were asked to select the “yummiest” from the fruits categorized as “just ok.” This process was then repeated for cookies. A child’s highest rated fruit and highest rated cookie were utilized for the subsequent RRV of food task. The RRV task was not performed if a child rated all three fruits or all three cookies as “yucky” (*n* = 1).

#### Child Anthropometry

Trained research staff measured children’s height and weight in duplicate. A portable stadiometer (Model 217; Seca Corporation) and digital scale (Model 843; Seca Corporation) were used to measure height and weight, respectively. A third height measurement was made if the first two differed by more than 1 cm, and a third was made for weight if the first two differed at all. BMI percentiles and BMIZ were calculated based on the 2000 CDC Growth Charts (US Centers for Disease Control and Prevention, 2002). Height and weight measurements were averaged for BMI percentile calculations; child age was calculated using children’s date of birth and the date that measurements were obtained. BMI percentiles <85th, ≥85th, and ≥95th classified children as having normal weight, overweight, and obesity, respectively. Due to space and time constraints, we were unable to measure children’s heights and weights immediately following the RRV task, therefore measurements were taken on the same day for most children in each classroom following RRV data collection. If participating children were absent when measurements were obtained, make-up days were scheduled through the end of data collection during each semester. On average, anthropometric data were collected 19 days after data collection, with a range of 0 to 64 days. Five children’s measurements were obtained two to 14 days prior to RRV data collection.

#### Questionnaires

Parents reported household food security status using the 18-item U.S. Department of Agriculture Household Food Security Module (HFSSM) (Coleman-Jensen et al., 2019). Children were considered FI if three or more of the 18 items were answered affirmatively and food secure if less than three of the 18 items were answered in the affirmative. Parent-reported reward sensitivity was measured using the child version (Blair, 2003; Blair et al., 2004) of the Behavioral Activation System (BAS) scale (Carver and White, 1994), a 13-item measure consisting of three subscales: drive (“My child goes out of his/her way to get something he/she wants”; four items), reward responsiveness (“It would excite my child very much to win a prize”; five items), and fun seeking (“My child acts on the spur of the moment”; four items). Response options range from 1 = “extremely untrue” to 7 = “extremely true.” A mean score of the three subscales was calculated to create a composite BAS scale (α = 0.88). Parents also self-reported their age, race/ethnicity, height/weight, education, marital status, income, employment, participation in the Supplemental Nutrition Assistance Program (SNAP) and the Supplemental Nutrition Program for Women Infants and Children (WIC) over the past 12 months, and child race/ethnicity. Child sex and date of birth were obtained from Head Start administrators.

### Statistical Analysis

The RRV outcomes analyzed included: the breakpoint for cookies (cookie Pmax), the breakpoint for fruit (fruit Pmax), the RRV of cookies as described previously, response rates for cookies and fruit, and post-task energy intake. To examine mean differences between cookies and fruit for the RRV outcomes and post-task energy intake, paired *t*-tests were used. To examine associations between RRV outcomes and continuous child characteristics (i.e., age in years, BMIZ, reward sensitivity), Pearson correlations were used. In addition to the total sample, correlations between RRV outcomes and continuous child characteristics were examined among children with normal weight (*n* = 76) and among children with overweight or obesity (*n* = 54). To examine differences in RRV outcomes by discrete characteristics (i.e., sex and age group), individual mixed models were conducted with the child characteristic as the independent variable and each RRV outcome as the dependent variable. *Post hoc* analysis with a Tukey’s adjustment for multiple comparisons was used to determine differences between 3-, 4-, and 5-year-olds. For mixed models with child sex as the independent variable, controlling for child age did not change results. For mixed models with child age as the independent variable, controlling for child sex did not change results. In addition, results regarding age and sex were similar after adjusting for BMIZ. Individual two-way mixed effects interaction models were used to examine the potential moderating role of age and sex on the association between the RRV of food and BMIZ. To examine differences in RRV outcomes by food security status, individual mixed models were conducted for each RRV outcome with household food security status as the independent variable. Results from models that included potential covariates (i.e., child sex, age, BMIZ, and SNAP participation) were similar, thus unadjusted models for differences in RRV outcomes by food security status are presented.

To assess differences in RRV outcomes by child weight status, individual mixed models were conducted. Initially, we examined differences in RRV outcomes for children with normal weight, overweight, and obesity. Based on trend-level differences for children with obesity compared to children with normal weight and children with overweight (0.05 < *p* < 0.10), and the observation that least squares means (LSmeans) responses for children with overweight were similar to children with normal weight, results are presented comparing children with obesity to children without obesity. One child was categorized as underweight (i.e., BMI percentile < 5%). Excluding this child did not change results, thus was retained in analyses examining the RRV of food by child weight status. In these models, two-way and three-way interactions between age, sex, and weight status on RRV outcomes were first tested. Interactions were not significant, and results did not change with the inclusion of age and sex as covariates, thus unadjusted main effects are presented.

Children’s pre-task hunger and the time between the end of lunch and the start of the RRV task were considered as covariates in all mixed models examining RRV outcomes as the dependent variable. These variables were not associated with RRV outcomes, child BMIZ, or household food security status (all *p* values > 0.05), and were not included in final models. Because children were sampled from eight Head Start centers, center location was included as a random effect in all mixed models. The caregiver survey asked parents to describe any medications that their child currently takes. One child was excluded from the analytic sample due to daily use of a dietary supplement for weight gain. An additional child was excluded because a parent sat with the child during the RRV task. Children with missing BMI data (*n* = 1) were excluded resulting in a final analytic sample of 130. An additional 20 participants were missing food security or SNAP data resulting in an analytic sample of 111 for analyses examining the relationship between FI and RRV outcomes. All analyses were performed using SAS Version 9.4 (SAS Institute Inc., Cary, NC, United States). Statistical significance was defined as *p* < 0.05 for all analyses.

## Results

### Sample Characteristics

Parent and child characteristics are shown in Table 1. The majority of parents were female (89%). On average, children were 4.49 ± 0.55 (Mean ± SD) years old and approximately half of the sample was female (52%). Children were predominantly white, non-Hispanic and from low-income, less educated households. Child BMI percentiles were 72.62 ± 24.98 (M ± SD), 25% of children were classified as having overweight, and 17% of children had obesity, which exceeds the national estimate for obesity prevalence among 2- to 5-year-old children (Hales et al., 2017). The prevalence of household FI was high (41%) and 77% of parents reported SNAP participation.

**TABLE 1:** Child and parent characteristics by child sex.

		**Total Sample (*n* = 130)**	**Male (*n* = 62)**	**Female (*n* = 68)**	

**Characteristics**	**N^2^**	***M* (SD)**	***M* (SD)**	***M* (SD)**	***p* value**
**Parent and household**					
Age^1^, years	106	31.49 (8.60)	32.08 (10.61)	30.94 (6.24)	0.76
Sex (%)	113				0.47
Female		89	91	86	
Male		11	9	14	
BMI	112	30.81 (9.95)	31.17 (11.71)	30.47 (8.07)	0.71
Race, *n* (%)	111				0.88
White		91	91	91	
Non-white		9	9	9	
Ethnicity (%)	107				0.57
Hispanic		5	94	96	
Non-Hispanic		95	6	4	
Education, *n* (%)	113				0.10
<High school degree		17	11	22	
High school degree		38	39	37	
Some college/technical school		32	37	27	
College degree		12	11	12	
Post-graduate training/degree		2	2	2	
Marital status, *n* (%)	113				0.28
Married		25	19	31	
Living with a partner		26	20	31	
Single		28	37	20	
Divorced/separated		19	20	19	
Widowed/other		2	4	0	
Annual household income, *n* (%)	93				0.20
<$10,000		32	35	30	
$10,000–$19,999		18	16	20	
$20,000–$29,999		24	19	28	
$30,000–$49,999		22	23	20	
≥$50,000		4	7	2	
Employment (%)	113				0.05
Unemployed		39	30	53	
Employed		61	70	47	
Food security status (%)	111				0.46
Food insecure (FI)		41	37	44	
Food secure		59	63	56	
SNAP participation (%)	112				0.18
Yes		78	72	83	
No		22	28	17	
WIC participation (%)	109				0.22
Yes		64	58	70	
No		36	42	30	
**Child characteristics**					
Age, years	130	4.49 (0.55)	4.52 (0.55)	4.47 (0.55)	0.61
Race, *n* (%)	109				0.50
White		84	87	82	
Non-white		16	13	18	
Ethnicity, non-Hispanic (%)	107				0.60
Hispanic		7	8	5	
Non-Hispanic		93	91	95	
BMI percentile	130	72.61 (24.98)	71.56 (24.68)	73.56 (25.40)	0.65

*SNAP, Supplemental Nutrition Assistance Program; WIC, Special Supplemental Nutrition Program for Women, Infants, and Children (WIC). Differences in participant characteristics by sex were examined using *t*-test for normal continuous variables, the Mann–Whitney *U* test for non-normal continuous variables, ^1^Chi-square test for binary variables, and Mantel-Haenszel chi-square for categorical variables.^2^Missing data for parent-reported characteristics because child participation was not contingent on parents completing a survey.*

Descriptive statistics for the study session (i.e., hunger, liking, and RRV outcomes) are presented in Table 2. Two children earned the maximum number of cookie and fruit portions (i.e., eight portions of each food), six children worked for cookies only, and 10 children worked for fruit only. On average, children worked 9.5 ± 6.1 min (mean ± SD) to access the foods. Paired *t*-tests showed that children had a higher breakpoint (5.0 ± 2.1 vs. 4.6 ± 2.2, *p* = 0.02) and had higher response rates (63.9 ± 30.0 vs. 58.7 ± 30.0, *p* = 0.03) for cookies compared to fruit. All RRV outcomes were correlated; RRV cookie was positively associated with cookie response rates (*r* = 0.31, *p* < 0.001) and negatively associated with fruit response rates (*r* = −0.41, *p* < 0.001). Cookie Pmax was positively associated with fruit Pmax (*r* = 0.50, *p* < 0.001), cookie response rates (*r* = 0.65, *p* < 0.001) and fruit response rates (*r* = 0.32, *p* < 0.001). Finally, fruit Pmax was positively associated with both cookie (*r* = 0.27, *p* < 0.01) and fruit (*r* = 0.57, *p* < 0.001) response rates. Additionally, RRV cookie, cookie and fruit Pmax, and response rates were positively associated with post-task energy intake (*r*’s = 0.17–0.62, *p* values < 0.05).

**TABLE 2:** Descriptive statistics for the RRV of food task (*n* = 130).

**Measure**	**Mean (SD)**	**Range**	
Time from lunch end to task start (min)	90.8 (17.5)	50.0–138.0	
Task duration, min	9.5 (6.1)	1–20	
**Hunger assessment**			
Pre-task	2.3 (0.8)	1–3	
Post-task	2.6 (0.7)	1–3	
**Liking assessment–cookies, *n* (%) yummy^1^**			*n* (%) chosen
Oreo minis^TM^	97 (75)	1–3	35 (27)
Fudge stripes minis^TM^	82 (63)	1–3	22 (17)
Circus animal^®^, cookies	99 (77)	1–3	73 (56)
**Liking Assessment–fruit, *n* (%) yummy^1^**			*n* (%) chosen
Red grapes	98 (75)	1–3	79 (61)
Mandarin oranges (canned)	75 (58)	1–3	35 (27)
Pineapple (canned)	66 (51)	1–3	16 (12)
**RRV task outcomes**			
RRV cookie	0.53 (0.2)	0–1	
Cookie Pmax	5.0 (2.1)	1–8	
Fruit Pmax	4.6 (2.2)	1–8	
Cookie response rate (responses/min)	63.9 (30.0)	0.0–132.2	
Fruit response rate (responses/min)	58.7 (30.0)	0.0–124.2	
**Post-task energy intake (kcals)**			
Total	161.0 (86.0)	0.0–403.8	
Cookies	132.7 (79.3)	0.0–371.8	
Fruit	28.3 (25.7)	0.0–108.1	
**Post-task energy intake (grams)**			
Total	77.4 (46.0)	0.0–183.8	
Cookies	26.1 (15.5)	0.0–71.5	
Fruit	51.2 (42.3)	0.0–143.7	

*Pmax, maximum schedule of reinforcement reached; RRV, relative reinforcing value. RRV cookie is the breakpoint for cookies in proportion to the total breakpoint for both cookies and fruit [RRV cookie = Cookie Pmax/(Cookie Pmax + Fruit Pmax)]. Calories (kcals) consumed were calculated using the post-weight and energy density (ED) of the food (kcals = ED × gram weight). Product label nutrition facts were used to determine the ED of study foods: Oreo minis^*TM*^ (4.83 kcals/g); Fudge stripes minis^*TM*^ (5.00 kcals/g); Circus animal^®^, cookies (5.20 kcals/g); red grapes (0.40 kcals/g); mandarin oranges (0.85 kcals/g); pineapple (0.85 kcals/g).*

*^1^Children indicated whether each food was yummy (1), just ok (2), or yucky (3).*

### Differences in the RRV of Food by Weight Status

**FIGURE 3:**
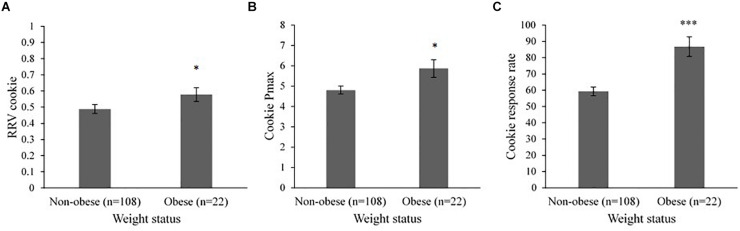
Mixed model analysis showing least squares means (LSmeans) ± SE differences in RRV cookie, cookie Pmax, and cookie response rates for children without obesity vs. children with obesity (*n* = 130). Head Start center (*n* = 8) was included as a random effect in all models. **(A)** Children with obesity (LSmean = 0.58 ± 0.04) had higher RRV cookie compared to children without obesity (LSmean = 0.49 ± 0.03, *p* = 0.04). **(B)** Children with obesity (LSmean = 5.86 ± 0.43) had higher cookie Pmax compared to children without obesity (LSmean = 4.81 ± 0.20, *p* = 0.03). **(C)** Children with obesity (LSmean = 86.78 ± 6.03) had higher cookie response rates compared to children without obesity (LSmean = 59.27 ± 2.72, *p* < 0.001). RRV, relative reinforcing value; Pmax, maximum schedule of reinforcement reached. RRV cookie is the breakpoint for cookies in proportion to the total breakpoint for both cookies and fruit [RRV cookie = Cookie Pmax/(Cookie Pmax + Fruit Pmax)]. Response rate is responses per minute. *p < 0.05, ***p < 0.001.

Mixed model analyses revealed differences in the RRV of cookies by child weight status (Figure 3). Children with obesity had higher RRV cookie [*F* (1, 121) = 4.28, *p* = 0.04], cookie Pmax [*F* (1, 121) = 4.95, *p* = 0.03], and cookie response rates [*F* (1, 121) = 17.27, *p* < 0.001] compared to children without obesity. There were no differences by weight status for fruit Pmax, fruit response rates, or post-task energy intake.

### The RRV of Food and Child Characteristics

Bivariate associations between the RRV of food and child characteristics are shown in Table 3. In the total sample, cookie Pmax (*r* = 0.17, *p* = 0.049), cookie response rates (*r* = 0.29, *p* = 0.001), and fruit response rates (*r* = 0.20, *p* = 0.024) were positively associated with child age. Cookie response rates were also positively associated with age among children with normal weight (*r* = 0.25, *p* = 0.03) and children with overweight/obesity (*r* = 0.32, *p* = 0.02); however, fruit response rates were only associated with age among children with normal weight (*r* = 0.36, *p* = 0.001). In addition, fruit Pmax was positively associated with age among children with normal weight only (*r* = 0.25, *p* = 0.03). Cookie response rates were positively associated with BMIZ (*r* = 0.26, *p* = 0.003) in the total sample, but this association was driven by children with overweight/obesity (*r* = 0.33, *p* = 0.02). Among children with overweight/obesity only, RRV cookie (*r* = 0.28, *p* = 0.04), post-task cookie intake (kcals; *r* = 0.28, *p* = 0.049), and total energy intake (kcals; *r* = 0.28, *p* = 0.04) were positively associated with child age; and RRV cookie was positively associated with BMIZ (*r* = 0.28, *p* = 0.04). RRV outcomes were not associated with reward sensitivity (*p* values > 0.05). As shown in Table 4, children aged 4 and 5 years responded at a faster rate for cookies [*F* (2, 120) = 8.08, *p* < 0.001] compared to 3-year-olds; and 4-year-olds had a higher breakpoint for cookies (cookie Pmax) [*F* (2, 120) = 3.70, *p* = 0.03] and responded at a faster rate for fruit compared to 3-year-olds [*F* (2, 120) = 3.62, *p* = 0.03]. Sex differences were not observed. Sex was evenly distributed across age groups and did not account for any of the significant associations between RRV and age (data not shown).

**TABLE 3:** Pearson’s correlation coefficients for RRV outcomes and child characteristics by weight status (*n* = 130).

	**Total sample (*n* = 130)**			**Normal weight (*n* = 76)**			**Overweight/obesity (*n* = 54)**		
	**Age**	**BMIZ**	**Reward**	**Age**	**BMIZ**	**Reward**	**Age**	**BMIZ**	**Reward**
	**(years)**		**Sensitivity**	**(years)**		**Sensitivity**	**years)**		**Sensitivity**
RRV cookie	0.02	0.13	0.10	–0.17	0.05	0.15	0.28*	0.28*	0.04
Cookie Pmax	0.17*	0.09	0.12	0.14	0.05	0.15	0.22	0.13	0.06
Cookie response rate (responses/min)	0.29**	0.26**	0.12	0.25*	0.00	0.18	0.32*	0.33*	0.04
Fruit Pmax	0.11	–0.03	–0.03	0.25*	0.01	–0.01	–0.08	–0.16	–0.08
Fruit response rate (responses/min)	0.20*	0.00	0.05	0.36**	–0.06	0.03	–0.02	–0.14	0.08
Post-task cookie intake (kcals)	0.10	–0.03	0.16	0.00	0.09	0.14	0.27*	–0.06	0.18
Post-task fruit intake (kcals)	0.14	–0.03	–0.02	0.17	0.02	–0.01	0.10	–0.07	–0.03
Post-task energy intake (kcals)	0.14	–0.01	0.14	0.05	0.09	0.13	0.28*	–0.07	0.16
Post-task cookie intake (grams)	0.11	–0.03	0.15	0.00	0.09	0.15	0.28*	–0.06	0.15
Post-task fruit intake (grams)	0.10	0.04	–0.02	0.12	–0.02	–0.05	0.06	–0.08	0.01
Post-task energy intake (grams)	0.13	0.02	0.03	0.11	0.02	0.01	0.14	–0.10	0.06

Pmax, maximum schedule of reinforcement reached. RRV, relative reinforcing value. RRV cookie is the breakpoint for cookies in proportion to the total breakpoint for both cookies and fruit [RRV cookie = Cookie Pmax/(Cookie Pmax + Fruit Pmax)]. Calories (kcals) consumed were calculated using the post-weight and energy density (ED) of the food (kcals = ED × gram weight). Product label nutrition facts were used to determine the ED of study foods: Oreo minis^*TM*^ (4.83 kcals/g); Fudge stripes minis^*TM*^ (5.00 kcals/g); Circus animal*^®^*, cookies (5.20 kcals/g); red grapes (0.40 kcals/g); mandarin oranges (0.85 kcals/g); pineapple (0.85 kcals/g). ^1^Behavioral Activation System (BAS) mean score. Response options, 1 = “Extremely untrue” to 7 = “Extremely true” [*n* = 106 due to missing parent-reported demographics (*n* = 17) and BAS scale (*n* = 7)]. *p ≤ 0.05, **p < 0.01. Bold significant correlations indicated with stars for ease of interpretation.

**TABLE 4:** LSmeans (SE) differences in the RRV of cookies and fruit by preschooler age and sex^1^.

	**Age (years)**			**Sex**	
	**3 (*n* = 28)**	**4 (*n* = 77)**	**5 (*n* = 25)**	**Male (*n* = 62)**	**Female (*n* = 68)**

**RRV outcome**	**LSmeans (SE)**	**LSmeans (SE)**	**LSmeans (SE)**	**LSmeans (SE)**	**LSmeans (SE)**
RRV cookie	0.49 (0.05)	0.51 (0.03)	0.52 (0.04)	0.50 (0.03)	0.52 (0.03)
Cookie Pmax	**4.07 (0.39)^a^**	**5.19 (0.23)^b^**	**5.36 (0.40)^a,b^**	4.97 (0.26)	5.00 (0.25)
Cookie response rate (responses/min)	**45.00 (5.39)^a^**	**68.07 (3.25)^b^**	**72.35 (5.70)^b^**	65.01 (3.83)	62.93 (3.65)
Fruit Pmax	4.06 (0.49)	4.95 (0.34)	4.88 (0.47)	4.80 (0.36)	4.77 (0.36)
Fruit response rate (responses/min)	**48.17 (6.34)^a^**	**65.46 (4.33)^b^**	**62.43 (6.23)^a,b^**	65.10 (4.78)	58.93 (4.69)
Post-task cookie intake (kcals)	122.36 (15.05)	133.26 (9.07)	142.54 (15.93)	143.52 (10.02)	122.83 (9.57)
Post-task fruit intake (kcals)	25.69 (6.32)	34.17 (4.80)	34.68 (6.01)	32.22 (5.08)	33.93 (4.98)
Post-task energy intake (kcals)	142.90 (16.25)	162.94 (9.80)	175.42 (17.20)	170.99 (10.90)	151.94 (10.40)
Post-task cookie intake (grams)	23.99 (2.94)	26.25 (1.77)	28.20 (3.11)	28.30 (1.96)	24.17 (1.87)
Post-task fruit intake (grams)	48.45 (9.76)	59.56 (7.05)	61.31 (9.38)	58.63 (7.50)	58.28 (7.36)
Post-task energy intake (grams)	71.54 (10.41)	85.08 (7.42)	89.22 (10.05)	86.67 (8.01)	82.09 (7.86)

*Pmax, maximum schedule of reinforcement reached. RRV, relative reinforcing value. RRV cookie is the breakpoint for cookies in proportion to the total breakpoint for both cookies and fruit [RRV cookie = Cookie Pmax/(Cookie Pmax + Fruit Pmax)]. Calories (kcals) consumed were calculated using the post-weight and energy density (ED) of the food (kcals = ED × gram weight). Product label nutrition facts were used to determine the ED of study foods: Oreo minis^TM^ (4.83 kcals/g); Fudge stripes minis^TM^ (5.00 kcals/g); Circus animal^®^, cookies (5.20 kcals/g); red grapes (0.40 kcals/g); mandarin oranges (0.85 kcals/g); pineapple (0.85 kcals/g).*

*^1^Mixed model analysis with Head Start location (*n* = 8) included as a random effect. LSmeans are least squares means ± SE. LSmeans that do not share superscripts differ by *p* < 0.05 according to Tukey’s adjusted *post hoc* comparisons. Significant differences were bolded for ease of interpretation.*

**FIGURE 4:**
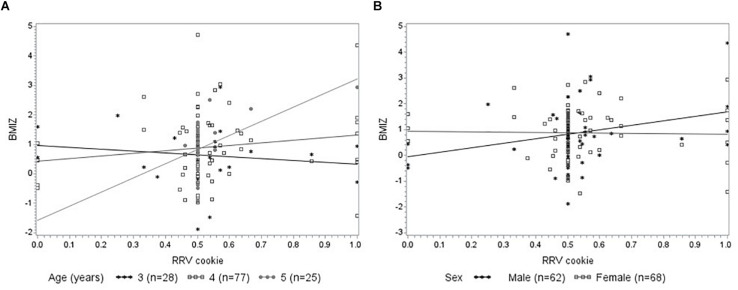
Mixed model analysis showing interactions between RRV cookie with child age and sex on BMIZ (*n* = 130). **(A)** There was an interaction between RRV cookie and child age [*F* (3, 123) = 2.86, *p* = 0.04] such that RRV cookie increased with increasing BMIZ for 5-year-olds (*p* = 0.02) but not for 3-year-olds (*p* = 0.52) or 4-year-olds (*p* = 0.12). **(B)** There was an interaction between RRV cookie and child sex [*F* (2, 126) = 3.25, *p* = 0.04] such that RRV cookie increased with increasing BMIZ for boys (*p* = 0.01) but not girls (*p* = 0.90). Head Start location (*n* = 8) was included as a random effect in all mixed models. BMIZ, BMI z-scores; Pmax, maximum schedule of reinforcement reached; RRV, relative reinforcing value. RRV cookie is the breakpoint for cookies in proportion to the total breakpoint for both cookies and fruit [RRV cookie = Cookie Pmax/(Cookie Pmax + Fruit Pmax)].

Next, we examined the potential moderating effect of child sex and age group on the relationship between RRV outcomes and BMIZ (Figure 4). There was an interaction between RRV cookie and child age [*F* (3, 123) = 2.86, *p* = 0.04] such that among 5-year-olds, RRV cookie increased with increasing BMIZ (*t* = 2.40, *p* = 0.02). RRV cookie was not associated with BMIZ among 3-year-olds (*t* = −0.65, *p* = 0.52) or 4-year-olds (*t* = 1.55, *p* = 0.12). In addition, there was an interaction between RRV cookie and sex on BMIZ [*F* (2, 126) = 3.25, *p* = 0.04] such that RRV cookie increased with increasing BMIZ for boys (*t* = 2.55, *p* = 0.01) but not girls (*t* = −0.13, *p* = 0.90). The interactions between RRV cookie and sex and RRV cookie and age on BMIZ did not change in models adjusting for age and sex, respectively (data not shown).

### The RRV of Food and Household Food Security Status

Least squares mean (LSmean) differences in RRV outcomes did not differ significantly by household food security status (*p* values > 0.05).

## Discussion

This study assessed differences in the RRV of HED (cookies) to LED (fruit) food by weight status and examined whether the RRV of food is associated with child characteristics (i.e., age sex, and reward sensitivity) and food security status in a sample of low-income children. Our results showed that children with obesity were more motivated to gain access to cookies relative to fruit compared to children without obesity. On average, children responded more for cookies and at a faster rate for cookies compared to fruit, which is consistent with prior studies (McCullough et al., 2017; Vervoort et al., 2017). Older children responded more for cookies and at a faster rate for both cookies and fruit, and higher BMIZ was associated with a faster rate of responding for cookies. Although proportional responding for cookies to fruit (i.e., RRV cookie) was not directly associated with BMIZ, the finding that child age and sex moderated the association between RRV cookie and BMIZ is novel. Our results extend the RRV of food and obesity literature to low-income preschool-age children and provide preliminary evidence for potential developmental and sex differences in this age group.

This is the first study to our knowledge to examine the RRV of food in low-income preschool-age children in a naturalistic setting (i.e., school), and is consistent with previous studies showing that higher RRV of food is associated with greater BMI in children from predominantly middle-to-upper-income, well-educated families (Temple et al., 2008; Rollins et al., 2014b; Kong et al., 2015; McCullough et al., 2017; Wong et al., 2019). While children with obesity worked harder for cookies compared to children without obesity, there was no difference in the reinforcing value of fruit by weight status. This is in contrast to McCullough et al. (2017), who also examined the RRV of cookies to fruit in a higher income sample, and found that the reinforcing value of fruit, but not cookies, differed by weight status such that children with overweight/obesity responded less for fruit compared to children with normal weight. This finding is similar to other studies in more advantaged samples showing that lean children find non-food alternatives (e.g., playing with toys) more reinforcing than children with overweight/obesity (Kong et al., 2015; Wong et al., 2019). The vastly different socioeconomic circumstance of our sample may explain this discrepancy. Greater exposure to a variety of healthy food options and/or cognitive stimulation (e.g., number of books, educational toys) in higher income households (Campbell et al., 2002; Rosen et al., 2020) may increase the salience of these alternative options, particularly for lean children. The RRV is somewhat dependent on the reinforcing value of the alternative choice that is available (Epstein et al., 2007), and it is important to point out that a sequential design, where only one food option is available at a time, is not a true choice paradigm. The RRV of fruit is likely to be lower in a concurrent design where the choice of fruit is directly compared to the choice of a cookie vs. the reinforcing value of fruit in a sequential design in which responding for cookies is measured separately from responding for fruit. Thus, the use of a concurrent design in this study, as opposed to a sequential design used in previous studies with preschoolers (McCullough et al., 2017; Wong et al., 2019), could also explain why responding for cookies, as opposed to fruit, drove differences in the RRV of cookie by weight status (McCullough et al., 2017).

As hypothesized, child age was associated with greater responding for cookies and faster response rates for both cookies and fruit, which is similar to prior research showing that children’s age is positively associated with total responses for food (Rollins et al., 2014b) and a higher breakpoint for monetary rewards (Chelonis et al., 2011). Though Rollins et al. (2014b) did not observe an association between age and response rates among a smaller sample (*n* = 30) of preschoolers, mean response rates were similar to our study (61.3 ± 30.0 vs. 55.8 ± 29.95), which is notable given these are the only two studies with young children having assessed response rates in the context of an RRV task. Partially supporting our hypotheses, cookie response rates, but not the breakpoint for cookies or RRV cookie, was positively associated with BMIZ. However, RRV cookie was positively associated with BMIZ among children with overweight/obesity and age moderated the association between RRV cookie and BMIZ such that greater proportional responding for cookies relative to fruit was associated with higher BMIZ among 5-year-olds, but not 3- or 4-year-olds. The moderating effect of age may indicate a developmental shift in which the RRV of food becomes a more salient predictor of obesity risk as children grow into early childhood, which could be attributed to older preschoolers having more experience with a broader variety of foods and/or more autonomy in making food-related decisions (Kininmonth et al., 2020). As previously suggested, it is also possible that younger children in our sample did not fully comprehend the task when presented with two foods concurrently (McCullough et al., 2017; Wong et al., 2019); or that they were more easily distracted or fatigued, both of which could mask the “true” breakpoint for one (or both) of the foods, thus affecting the reliability of concurrent reinforcement schedules in this age group (Rollins et al., 2014b).

Findings that the RRV of food varies by sex have been mixed in both animal and human studies with adults (Van Haaren et al., 2001; Roth et al., 2005; Goldfield and Lumb, 2008). In contrast to what was hypothesized, the current study did not observe differences in RRV outcomes by sex. Previous studies with children and adolescents show that boys make more responses for food than girls (Rollins et al., 2014b; Vervoort et al., 2017). One potential explanation for this discrepancy is differences in how food rewards are presented to children after earning each reward during the RRV task. Rollins et al. (2014b) allowed children to eat food portions earned throughout the task, but in the current study children received food portions as they were earned but were not allowed to eat the foods until after the task was completed. The current study’s methods to assess RRV are similar to the delay of gratification protocol, a commonly used measure of self-control that measures the ability to wait for larger quantities of food (Schlam et al., 2013). A study conducted by Gearhardt et al. (2017) that used methods comparable to our study reported a positive association between RRV and delay of gratification, and performance on the RRV task was similar between boys and girls. Thus, conducting the RRV task in such a way that children must delay a smaller, immediate reward vs. working longer to obtain a larger, delayed reward may be confounded by delay of gratification. Sex differences in the motivation to obtain food rewards may be more apparent in the RRV task when children are allowed to eat each portion of food as earned throughout the task and elements of self-control are not introduced. This nuanced difference in the assessment of RRV may also explain why the current study did not replicate the positive association between reward sensitivity and RRV observed by Rollins et al. (2014b). We did find, however, an interaction between RRV cookie and child sex on BMIZ, with an association between greater proportional responding for cookies to fruit and higher BMIZ among boys but not girls. In contrast, Gearhardt et al. (2017) showed an association between higher RRV and overweight in girls, but not boys, among 7- to 10-year-old low-income children. These incongruent findings may be due to the difference in child age between the two studies and/or the use of toys rather than a LED food as the alternative reinforcer in the study conducted by Gearhardt et al. (2017). Given the limited evidence, more research is needed examining sex differences, how the RRV of food develops in relation to obesity risk across childhood, and whether temperamental traits other than reward sensitivity contribute to individual differences in the RRV of food.

Approximately 41% of low-income families in our sample reported household FI. Different from previous research in adults showing that FI is associated with the RRV of HED snack foods (Crandall and Temple, 2018; Crandall et al., 2020), we did not observe a significant association between household FI and the RRV of HED food. In both adult studies conducted by Crandall and colleagues, participants worked for a non-food alternative (e.g., reading), whereas the alternative reinforcer in our study was a LED food (i.e., fruit). The fruits used in our study, which were well-liked and familiar to children, may have been too reinforcing to see an effect of FI on the RRV of HED food. Similarly, a more desired novel HED food may be required to elicit a FI effect. Future research with children should examine the relationship between FI and the RRV of HED food using a non-food alternative (e.g., toys) and/or using palatable foods such as candy that are typically not allowed in the childcare setting (e.g., candy).

It is important for future research to design and evaluate evidence-based strategies that aim to simultaneously reduce the RRV of HED food and increase the reinforcing value of healthier alternatives. Previous studies with young children shed light on approaches that could be used to increase the reinforcing value of LED foods so that they can compete with more rewarding HED foods. A small, randomized trial with infants showed that a music enrichment program increased music reinforcement and reduced the RRV of food (Kong et al., 2016), suggesting that frequent exposure to a pleasurable non-food alternative has the potential to reduce food reinforcement. Repeated exposure of small tastes of vegetables increases liking and intake of those vegetables (Anzman-Frasca et al., 2012); however, research has not tested whether repeated exposure of healthier foods can sensitize, or increase, their reinforcing value among children. Repeated consumption of a food over a period of days or weeks can lead to a decline in the pleasantness of that food (i.e., monotony) (Hetherington et al., 2002). Alternating between a variety of LED foods within a repeated exposure intervention, or pairing target foods with positive stimuli (Vervoort et al., 2017) could be used to avoid a monotony effect. Although FI was not associated with the RRV of food in our sample, it is important to keep in mind that increasing access to both healthier foods and stimulating activities will pose a greater challenge to low-income families (Carr and Epstein, 2020). Given the RRV of HED food was driven by cookies (and not fruit) in the current study, research should also test if decreasing access to HED foods or if decreasing children’s exposure to a variety of HED foods can reduce food reinforcement. However, overt restriction of specific foods, which has been shown to increase their RRV (Rollins et al., 2014a), should be avoided.

There are several strengths and limitations to consider. First, the present study was cross-sectional, thus causality cannot be inferred. Little is known about early developmental changes in motivational processes such as RRV and how such changes might link to variations in obesity risk across childhood. Though our sample was large enough to examine individual differences in the RRV of food in relation to child age, an important next step is longitudinal research that is able to assess RRV in the same children over time. Second, there were minor inconsistencies in delivery of the RRV protocol. For example, rather than providing a standardized meal prior to the task we relied on children’s typical Head Start provided lunch, which differs from day to day. Further, children’s height and weight measurements were not obtained on the same day that children participated in the RRV task. We cannot rule out the possibility of bias from such inconsistencies; however, this type of measurement error is often biased toward a null finding (Hammer et al., 2009). This is one of a few studies to conduct the RRV of food task outside of a laboratory setting and to examine the RRV of food among low-income children, which is important given economically disadvantaged children are at a greater risk of obesity and often a harder to reach population. On the other hand, the current sample was predominantly white, low-income, and rural. While more research is needed in low-income populations, future research would benefit from larger, more diverse samples in order to better generalize findings. Finally, it is difficult to disentangle delay of gratification as a potential confounder given the current study did not allow children to consume their food rewards until the task was completed. In addition to replicating the current study’s findings using methods that do not overlap with delay of gratification, future research should examine the RRV of HED food relative to a non-food alternative in a sample of low-income preschoolers.

In summary, using a choice paradigm to study the RRV of food, the current study found that low-income children with obesity responded more and at a faster rate for cookies and had higher proportional responding for cookies to fruit compared to children without obesity. It will be important to determine whether increasing access to a variety of LED foods or non-food alternatives while decreasing access to a variety of HED foods can facilitate healthy decision making in children (Carr and Epstein, 2020). In our sample of preschoolers, a period in which several developmental milestones are reached as children go from 3 to 5 years, we observed both age and sex differences. Among older children and boys, children with greater proportional responding for cookies to fruit tended to have higher BMI z-scores. The breakpoint for cookies, but not fruit, was higher in children with obesity compared to children without obesity, suggesting that the greater RRV of cookies in children with obesity was driven by greater motivation to access cookies rather than a lower motivation to access fruit. These findings highlight the need to identity approaches to reduce the RRV of HED foods among low-income children with obesity. Household food security status was not associated with RRV outcomes. Research is needed to identify and understand other home environment characteristics that influence the development of individual differences in food reinforcement in order to inform the development of primary obesity prevention programs for low-income children.

## Data Availability Statement

The raw data supporting the conclusions of this article will be made available by the authors, without undue reservation.

## Ethics Statement

The studies involving human participants were reviewed and approved by Office for Research Protections at the Pennsylvania State University, United States. Written informed consent to participate in this study was provided by the participants’ legal guardian/next of kin.

## Author Contributions

SGE and JSS designed the research and had primary responsibility for final content. SGE conducted the research, analyzed the data, and wrote the first draft of the manuscript. MEM assisted with statistical analysis. KLK, JLT, MEM, and JSS interpreted the data and revised the manuscript. All authors read and approved the final manuscript.

## Conflict of Interest

The authors declare that the research was conducted in the absence of any commercial or financial relationships that could be construed as a potential conflict of interest.
